# LETTER TO THE EDITOR Protocols for the Surgical Management of Burn Injury: Has Their Development Been Worthwhile?

**Published:** 2009-09-15

**Authors:** John E. Greenwood

**Affiliations:** Burns Unit, Royal Adelaide Hospital, North Terrace, Adelaide, South Australia

Dear Sir,

Surgical protocols were introduced by the Adult Burn Service, Royal Adelaide Hospital, in early 2005,^1^ documenting both instructions and techniques. Underlying these protocols is the need for expeditious burn excision. Where lower airway inhalation injury coexists, there appears to be a “surgical window” that opens at the time of the burn injury and closes 48 to 72 hours later. Although resuscitation is ongoing, the respiratory tract appears robust to early surgery. Following resuscitation, in patients with inhalation injury, the respiratory tract “goes off” as chemical pneumonitis and adult respiratory distress syndrome develop around the 48- to 72-hour point. If burn remains at this stage, the patient is often too unwell for aggressive surgery. By the time the respiratory tract has recovered sufficiently, retained burn eschar starts degrading and is bacterially contaminated. Systemic problems, especially renal, ensue. Inotrope medication is most likely to be necessary during this time. This has the potential (at doses > 30 µg/min noradrenaline) to convert superficial burns and donor sites to full-thickness injuries and critically impair the developing blood supply to skin grafts and Integra, resulting in loss.

The burns to be excised and proposed donor sites are tumesced subcutaneously with a solution of 1:500,000 adrenaline in 0.9% saline. Blood transfusion is uncommon and excisions of more than 80% total body surface area (TBSA) can be performed on admission. This is in keeping with published literature.^2^

The protocols have proved invaluable. As stepwise instructional aids for new registrars involved in burn care on-call and preparation for specialist examinations, no published alternative exists. Other units have used them as templates for their own protocol development.

They have allowed considered evaluation of new techniques and dressings against an established “gold standard” of treatment enabling accurate comparison. They have safeguarded the use of expensive materials (eg, Integra), incorporated into well-constructed and transparent algorithms, from administrative and budgetary scrutiny.

Some changes have been necessary (and anticipated) in the past 4 years. Transcyte was our primary face burn dressing in burns greater than 20% TBSA; this material is now (sadly) unavailable. Face care on the Burn Unit can be managed simply with soft paraffin and meticulous care, but in ICU, the siting of the endotracheal and nasogastric/feeding tubes, with intraoral and airway burns, leads to fluid leakage from nares and oral commissures, risking infection of facial burns. Aquacel Ag, a silver-impregnated alginate, adheres well to facial exudate needing no fixation. It can be easily removed/refreshed and easily fashioned into a face mask.

The Skin Engineering Laboratory previously supplied a noncultured suspension of keratinocytes and melanocytes, analogous to C3's Re-Cell™, to hasten re-epithelialization of donor sites and meshed split-skin graft interstices. However, a randomized, placebo-controlled trial failed to show any ability to expedite reepithelialization,^3^ forcing removal from use.

The failure of both Victorian and New Zealand Cadaver Skin banks to reliably provide allograft for patients with major burn injury led to its removal from the protocols. Biobrane works just as well in temporizing the debrided burn wound.^4^

Protocol development has thus been worthwhile on many levels; however, regular audit must follow and changes are inevitable. They remain available to anyone on application.
